# Current progress and emerging trends of car-based immunotherapy for hepatocellular carcinoma: a data-driven atlas from multidatabase integration

**DOI:** 10.3389/fimmu.2026.1778197

**Published:** 2026-04-22

**Authors:** Hao Ying, Yuhao Pan, Xingyu Zhu, Qijia Xuan

**Affiliations:** 1 Department of Oncology, the Fourth Affiliated Hospital of School of Medicine, and International School of Medicine, International Institutesof Medicine, Zhejiang University, Yiwu, China; 2 Department of Laboratory Medicine, The First Hospital of China Medical University, Shenyang, China

**Keywords:** CAR-based immunotherapy, chimeric antigen receptors (CARs), hepatocellular carcinoma, immunotherapy, multidatabase

## Abstract

**Background:**

Hepatocellular carcinoma (HCC) is a highly prevalent and lethal malignancy worldwide, with its incidence steadily rising over recent decades. Conventional treatments, including chemotherapy, surgery, immunotherapy, Targeted Therapy and radiotherapy, offer limited therapeutic benefit. Chimeric antigen receptor (CAR)-based immunotherapy has emerged as a promising strategy for HCC, yet significant challenges remain. This study aims to provide a comprehensive overview of the current landscape and future prospects of CAR-based immunotherapy in HCC.

**Methods:**

On September 30, 2025, we conducted a systematic literature search in the Web of Science Core Collection (WoSCC) and Scopus databases, targeting publications from 2016 to 2025. Full records and cited references were extracted and screened. Data-driven analyses and visualizations were performed using CiteSpace, Microsoft Excel 2019, VOSviewer, and R software.

**Results:**

A total of 5,210 publications were included in the analysis. The number of studies in this field has increased sharply since 2019 and is projected to continue rising. China, particularly its universities, leads global research in this area. Most influential studies were concentrated around 2019. Research hotspots have shifted from traditional pharmacology toward gene regulation and the tumor microenvironment (TME). Collaboration networks among authors, countries, and institutions indicate a growing trend toward multidisciplinary cooperation.

**Conclusion:**

CAR-based immunotherapy for HCC remains in its early stages of development. Future research should prioritize enhancing therapeutic efficacy and safety, fostering international collaboration, and promoting interdisciplinary approaches to improve clinical outcomes for HCC patients.

## 1 Introduction

Hepatocellular carcinoma (HCC) is the dominant form of primary liver cancer, accounting for over 90% of cases, and remains one of the leading causes of cancer-related mortality worldwide, ranking third globally (1, 2). Because the disease typically develops without overt clinical symptoms and lacks reliable early diagnostic markers, nearly 70–80% of patients are diagnosed at intermediate or advanced stages, when curative treatment options are limited. As a result, overall prognosis remains poor, with the heaviest disease burden observed in East Asia and sub-Saharan Africa (3).

The therapeutic landscape of HCC currently includes surgical resection, liver transplantation, local ablative techniques, transarterial chemoembolization (TACE), radiotherapy, systemic chemotherapy, molecularly targeted agents, and immune checkpoint inhibitors (ICIs) (4, 5). However, survival benefits in advanced-stage disease remain modest. This limited efficacy is largely attributable to marked intratumoral heterogeneity, impaired hepatic functional reserve, and the frequent emergence of drug resistance. Consequently, the five-year overall survival rate remains below 20% (6, 7). Although immune checkpoint blockade targeting PD-1/PD-L1 and CTLA-4 has improved outcomes in a subset of patients, response rates remain low, and immune escape mechanisms together with immune-related toxicities continue to restrict long-term clinical benefit (8, 9).

Chimeric antigen receptor (CAR)–based cellular immunotherapy has fundamentally reshaped the field of cancer immunotherapy. Through genetic engineering, immune effector cells are endowed with antigen-specific recognition capacity, enabling potent tumor cell killing in a major histocompatibility complex–independent manner (10, 11)The clinical success of CAR-T cell therapy in hematological malignancies represents a major milestone. In 2017, the US Food and Drug Administration approved the CD19-targeted CAR-T products Kymriah and Yescarta for the treatment of relapsed or refractory B-cell malignancies, establishing CAR therapy as a viable clinical modality (12, 13). These achievements have prompted increasing efforts to adapt CAR-based strategies for solid tumors, including HCC (14).

In contrast to hematological cancers, solid tumors pose distinct biological and therapeutic barriers to CAR-based interventions (15). HCC is characterized by a highly immunosuppressive tumor microenvironment enriched in myeloid-derived suppressor cells (MDSCs) and tumor-associated macrophages (TAMs). These immunosuppressive populations secrete cytokines such as TGF-β and IL-10, which collectively limit CAR cell trafficking, proliferation, and cytotoxic function within the tumor milieu (16, 17). Moreover, the identification of suitable target antigens remains a central challenge. Optimal CAR targets must be selectively and robustly expressed on tumor cells while exhibiting minimal expression in normal liver tissue. To date, glypican-3 (GPC3) and alpha-fetoprotein (AFP) remain the most extensively studied HCC-associated antigens (18, 19). Additional constraints, including the liver’s intrinsic immune-tolerant environment, treatment-related toxicities, and the high cost of CAR therapies, further limit their broad clinical application.

Despite these challenges, accumulating evidence supports the therapeutic promise of CAR-based approaches in HCC. Preclinical models and early-phase clinical studies have demonstrated that CAR-T cells targeting antigens such as GPC3 and CD133 can effectively eliminate HCC cells, with acceptable safety profiles and preliminary signs of clinical activity in selected patients (20, 21). Globally, the number of CAR-related clinical trials in HCC continues to increase, reflecting sustained momentum in the field. Importantly, research efforts have progressed beyond early single-antigen strategies toward more sophisticated paradigms, including multi-target CAR designs, logic-gated CAR architectures, CAR-NK cell platforms, combination therapies, and strategies aimed at remodeling the tumor microenvironment (22).

Amid this landscape, a systematic evaluation of CAR-based immunotherapy research in HCC is needed. Bibliometric analysis offers a data-driven approach to map research activity, track thematic evolution, and reveal international collaboration patterns. This study integrates data from the Web of Science Core Collection, Scopus, and PubMed, using CiteSpace, VOSviewer, and R-based methods to provide a comprehensive overview. By highlighting current trends and emerging directions, it aims to inform future mechanistic studies and support clinical translation.

## 2 Materials and methods

### 2.1 Data sources and search strategy

**Figure 1:**
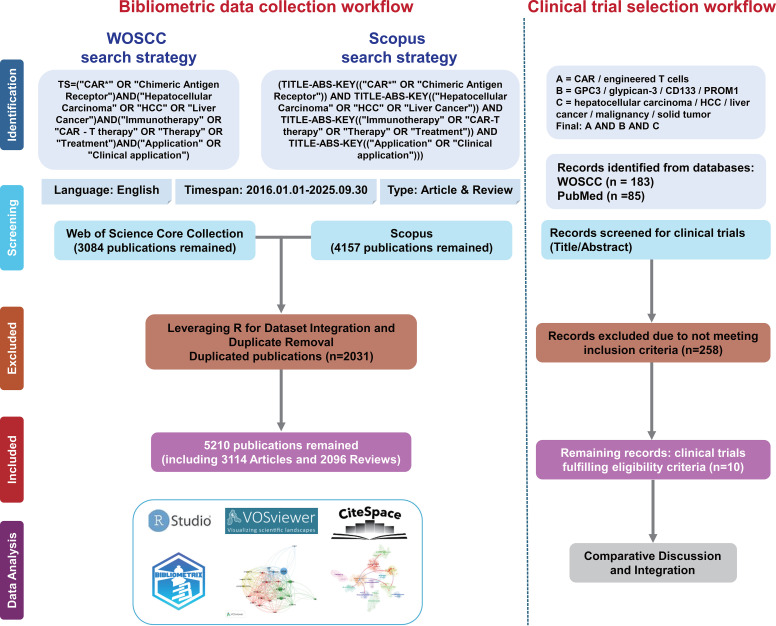
Flowchart of multi-database inclusion/exclusion criteria.

Literature published between 2016 and 2025 was retrieved from the Web of Science Core Collection (WoSCC) and Scopus on September 30, 2025. The search strategy was: TS=(“CAR*” OR “Chimeric Antigen Receptor”) AND (“Hepatocellular Carcinoma” OR “HCC” OR “Liver Cancer”) AND (“Immunotherapy” OR “CAR-T therapy” OR “Therapy” OR “Treatment”) AND (“Application” OR “Clinical application”).Only English-language articles and reviews were included. Full records and cited references were exported to capture metadata on authorship, publication year, journal, citations, and institutions. Initial retrieval yielded 3,084 records from WoSCC and 4,157 from Scopus. After automated deduplication and manual verification, 5,210 unique records (3,114 articles and 2,096 reviews) were retained for analysis (**Figure 1**). All procedures were independently performed by three researchers, with disagreements resolved by discussion (**Supplementary Table 1**).

For the clinical trial component, we additionally developed a separate search strategy and predefined inclusion and exclusion criteria to identify eligible studies on CAR-based immunotherapy targeting representative HCC-associated antigens, including GPC3 and CD133/PROM1 (**Supplementary Table 2**). The search was performed in WoSCC and PubMed. After title and abstract screening, 10 clinical trials fulfilling the eligibility criteria were retained for comparative discussion and integration (**Supplementary Table 3**). All screening procedures were independently completed by three researchers, and any disagreements were resolved through discussion and consensus.

### 2.2 Data analysis and visualization

Data were imported into CiteSpace, VOSviewer, R (Bibliometrix), and Excel for analysis and visualization. Excel was used to examine annual publication trends and citation counts. Bibliometrix (R) analyzed international collaboration networks. VOSviewer mapped contributions of countries, institutions, authors, and journals, as well as keyword co-occurrence and co-citation networks (23). CiteSpace was used for co-citation and keyword clustering analyses (24).

### 2.3 Analytical Tools and Parameters

CiteSpace (v6.3.R2) was configured with a 2016–2025 time span, 1-year slices, node types including Reference, Author, Institution, Country, and Keyword, g-index (k = 25) thresholds, and Pathfinder pruning. Clusters were labeled using the log-likelihood ratio (LLR), with modularity (Q) and silhouette scores assessing quality. VOSviewer (v1.6.20) employed full counting, analyzed Keywords Plus, and included terms occurring ≥5 times. R (v4.3.1) used Bibliometrix functions biblioAnalysis(), networkPlot(), and thematicEvolution(). Excel 2019 supported data organization and annual publication statistics.

### 2.4 Quality control and stratified assessment

Because bibliometric studies are especially sensitive to noise introduced during data curation, we implemented an additional quality-control procedure before conducting the final analyses. To improve the overall reliability of the dataset, publication types with limited peer review or comparatively low academic rigor—including conference abstracts, letters, and editorials—were excluded at the screening stage. Only original articles and review papers were retained. This restriction was intended to reduce heterogeneity attributable to publication format and, in turn, to strengthen the interpretability of the bibliometric patterns derived from the final corpus.

Beyond document-type filtering, we established a three-level quality appraisal framework covering study design transparency, data completeness, and conclusion reliability (Grade A, high quality; Grade B, moderate quality; Grade C, low quality) (**Supplementary Table 4**). To test the reproducibility of this framework, three researchers independently and blindly evaluated a random 20% sample of the included literature (n = 1,042). Inter-rater agreement was assessed using Fleiss’ kappa coefficient. The overall kappa value was 0.88 (95% CI: 0.83-0.93), indicating excellent agreement among the three assessors. Within the sampled studies, 77.9% were classified as high quality (Grade A), whereas only 4.3% were assigned to Grade C because of evident methodological weaknesses (**Supplementary Table 5**). Taken together, the predominance of Grade A studies and the very high level of agreement between evaluators provide strong support for the robustness of the analytical framework and, by extension, for the reliability of the results reported in this study.

During this process, we also noted that nearly all highly cited publications fell within the Grade A category. Although this observation was incidental rather than hypothesis-driven, it suggests that the major developmental trends in this field have been shaped primarily by higher-quality evidence, while the potential influence of lower-quality studies on the overall bibliometric associations appears to be limited.

## 3 Results

### 3.1 Publication trends and geographic distribution

**Figure 2:**
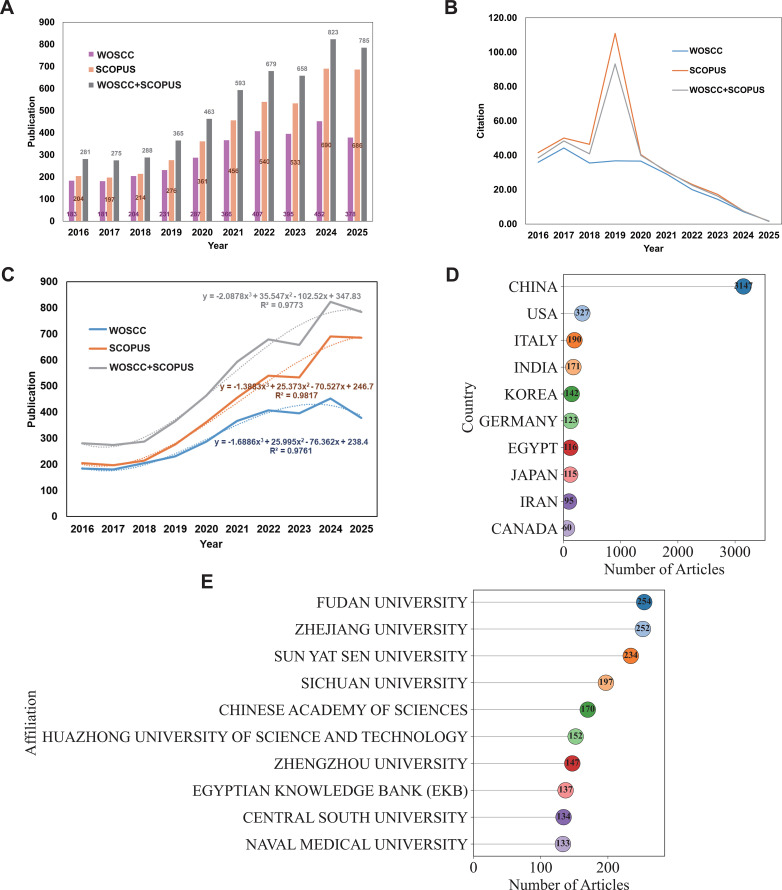
Publication Trends and Geographical Distribution. **(A)** Annual publication trends of the research (WOSCC, SCOPUS, WOSCC+SCOPUS); **(B)** Annual citation analysis (WOSCC, SCOPUS, WOSCC+SCOPUS); **(C)** Fitted curves of publication output (WOSCC, SCOPUS, WOSCC+SCOPUS); **(D)** Top ten countries by publication output (WOSCC+SCOPUS); **(E)** Top ten institutions by publication output (WOSCC+SCOPUS).

Between 2016 and 2018, annual publications on CAR-based immunotherapy in HCC remained relatively stable across WoSCC and Scopus. Starting in 2019, output increased sharply, reaching 823 articles in 2024, with 785 published by September 2025. Across the entire period, Scopus consistently indexed more articles than WoSCC, reflecting differences in database coverage (**Figure 2A**). Annual citation trends were consistent across databases, peaking for studies published around 2019, which exerted the greatest impact on the field (**Figure 2B**). In subsequent years, publication output continued to rise, but average citations per article declined, indicating a diversification of research focus and an expansion phase of the field. Publication counts from both databases exhibited strong positive correlations with year (R² = 0.976–0.981), suggesting that 2025 is likely to surpass previous records and confirming a period of rapid growth in CAR-HCC research since 2019 (**Figure 2C**). Geographically, China led the field with 3,147 publications, followed by the United States (327) and Italy (190) (**Figure 2D**, **Table 1**). Despite contributing over 60% of the global literature, China’s share of internationally collaborative papers remained lower than that of other leading countries, highlighting strong domestic output but limited cross-border collaboration.

**Table 1:** Top ten countries by publication output, including SCP and MCP (WOSCC+SCOPUS).

Country	Articles	Articles %	SCP	MCP	MCP %
CHINA	3147	60.4	2971	176	5.6
USA	327	6.3	271	56	17.1
ITALY	190	3.6	157	33	17.4
INDIA	171	3.3	154	17	9.9
KOREA	142	2.7	126	16	11.3
GERMANY	123	2.4	88	35	28.5
EGYPT	116	2.2	100	16	13.8
JAPAN	115	2.2	100	15	13
IRAN	95	1.8	73	22	23.2
CANADA	60	1.2	47	13	21.7

Institutional analysis revealed that Fudan University was the most productive, with 254 publications, followed closely by Zhejiang University (252) and Sun Yat-sen University (234). Sichuan University (197) and the University of Chinese Academy of Sciences (170) also ranked among the top five, underscoring the dominant role of Chinese institutions in advancing CAR-HCC research (**Figure 2E**).

### 3.2 Author, institutional, and national research output

**Figure 3:**
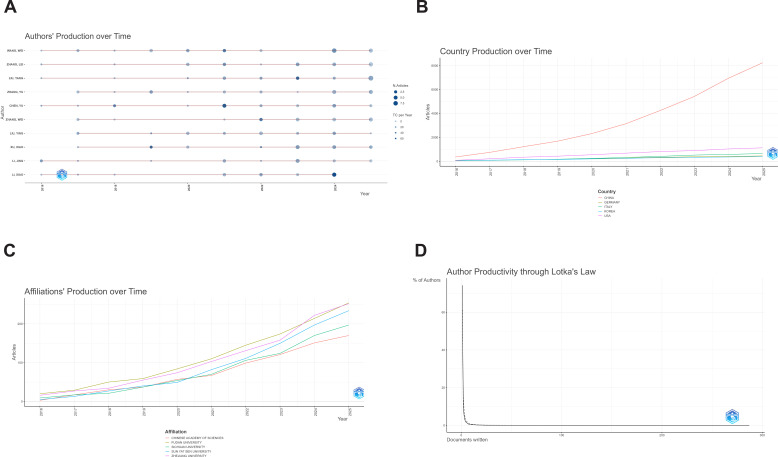
Analysis of author, institutional, and national output: **(A)** Annual Author Output Analysis (WOSCC+SCOPUS); **(B)** Annual Country Output Analysis (WOSCC+SCOPUS); **(C)** Annual Institutional Output Analysis (WOSCC+SCOPUS); **(D)** Author Output Lotka’s Law (WOSCC+SCOPUS).

In systematically analyzing the landscape of research contributors in this field, we examined authors, institutions, and countries, complemented by Lotka’s law to validate the distribution of author productivity. A core group of authors began to emerge around 2016 and has remained consistently active. Among them, CHEN YU, despite publishing only 20 papers (NP = 20), achieved the highest single-paper impact with 837 total citations, an h-index of 13, and an m-index of 1.3, represented by the largest bubble in **Figure 3A**, indicating a high citation density. WANG WEI (NP = 27, TC = 557, h-index = 12) and ZHANG LEI (NP = 23, TC = 408, h-index = 10) exemplify highly productive and stable contributors, with steadily increasing annual outputs and cumulative academic influence. LIU YANG (NP = 22, TC = 539, g-index = 22), though slightly less prolific, displays a higher concentration of highly cited publications, reflecting a strong focus in knowledge contribution. FAN JIA (TC = 532, NP = 13), while attaining considerable total citations, exhibits a lower h-index (9) and m-index (0.9), suggesting that impact is primarily driven by a few highly cited works. LI MIN (NP = 14, h-index = 11, m-index = 1.375) and CHENG HONGWEI (NP = 12, h-index = 9, m-index = 1.125) demonstrate relatively high academic efficiency despite limited output. Overall, most core authors began publishing between 2016 and 2018, with a marked acceleration after 2020, indicating that the field has entered a phase of rapid development (**Table 2**).

**Table 2:** Top 10 authors by publication output (web of science core collection + scopus).

Author	h_index	g_index	m_index	TC	NP	PY_start
CHEN YU	13	20	1.3	837	20	2016
WANG WEI	12	23	1.2	557	27	2016
LI MIN	11	14	1.375	464	14	2018
LIANG PING	11	11	1.1	371	11	2016
LIU YANG	11	22	1.1	539	22	2016
LI JUN	10	11	1	300	11	2016
ZHANG LEI	10	20	1	408	23	2016
ZHANG YU	10	19	1.111	364	22	2017
CHENG HONGWEI	9	12	1.125	302	12	2018
FAN JIA	9	13	0.9	532	13	2016

At the country level, China has experienced a rapid rise since 2010, with annual publications increasing from fewer than 500 to nearly 6,000 by 2025, reflecting exponential growth and consolidating its leading position globally. The United States has maintained steady growth, producing roughly 800–1,200 papers annually and ranking second; however, the gap with China continues to widen. Countries such as Germany, Italy, and South Korea contribute relatively fewer publications, generally ranging between 100 and 300 per year, with gradual growth, suggesting limited participation or focus on niche areas. This pattern has resulted in a bipolar structure centered on China as the core, with the U.S. as a major collaborator and other developed nations serving peripheral roles (**Figure 3B**).

At the institutional level, the Chinese Academy of Sciences has consistently led the field since 2005, surpassing 200 publications annually by 2025, representing the most significant contribution. Among universities, Fudan University and Sichuan University have rapidly advanced since 2015, with steady increases in annual output, while Zhejiang University has shown a marked rise from 2023 to 2025, indicating emerging research momentum. Sun Yat-sen University, although a later entrant, has also exhibited an upward trend in recent years. Overall, leading Chinese universities and national research institutions collectively form the primary innovation engine in this field (**Figure 3C**).

Analysis of author productivity distribution reveals that over 80% of authors published only a single paper, with the proportion declining sharply for two or more publications. Only a small fraction of authors, such as CHEN YU and WANG WEI, published more than 20 papers, consistent with the “inverse square law” described by Lotka’s law (**Figure 3D**). This distribution highlights that knowledge production is highly concentrated among a few prolific authors, while the majority engage sporadically, further corroborating the nonuniformity of research activity and the core–periphery structural characteristic of the field.

### 3.3 Keyword analysis

**Figure 4:**
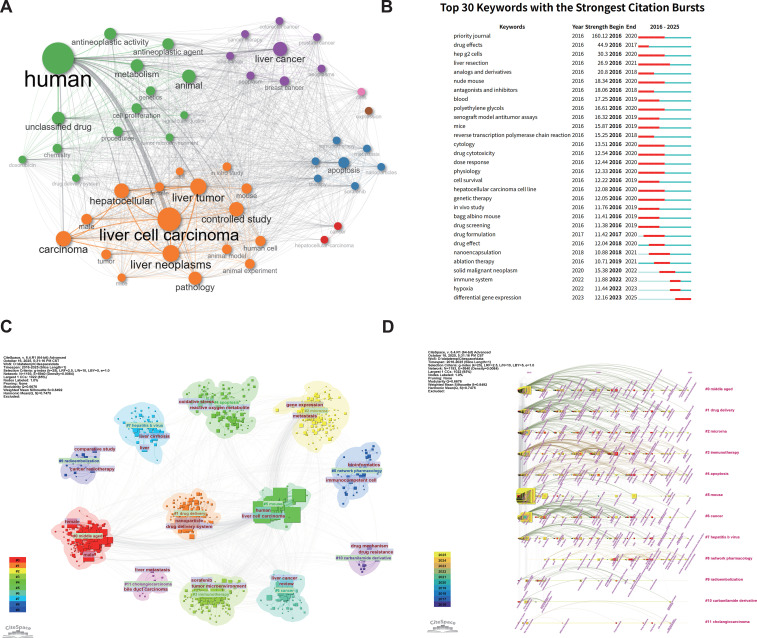
Keyword analysis: **(A)** Co-occurrence network of high-frequency keywords (WOSCC+SCOPUS); **(B)** Top 30 keywords by burst strength (WOSCC+SCOPUS); **(C)** Keyword cluster map (WOSCC+SCOPUS); **(D)** Keyword timeline map (WOSCC+SCOPUS).

To explore the development and future directions of CAR-based immunotherapy in HCC, we performed keyword network analysis using CiteSpace and R-bibliometrix. The keyword co-occurrence network was visualized using the bibliometrix package in R (**Figure 4A**). The network layout was set to ‘Automatic’, and the Louvain algorithm was employed for clustering. To ensure the robustness of the network, we applied filtering options by removing isolated nodes and setting the minimum number of edges to 2. The network size was limited to the top 50 most frequent keywords for clarity, with a repulsion force of 0.5 to optimize node separation. Co-occurrence analysis revealed four main clusters (**Figure 4A**). The orange cluster, including “liver cell carcinoma” and “pathology,” reflects clinical and pathological research. The green cluster, with “antineoplastic activity” and “animal model,” represents drug development and metabolic studies. The blue and purple clusters, featuring “apoptosis” and “liver cancer,” highlight molecular mechanisms and cross-tumor research. Overall, HCC research shows a multi-thematic pattern integrating clinical and basic studies.

We then analyzed keyword bursts and identified the top 30 high-burst keywords (**Figure 4B**). Early bursts (2016–2019), such as “drug effects” and “cytotoxicity,” indicate an initial focus on pharmacology. Mid-stage bursts (2020–2022), including “gene therapy” and “drug delivery,” highlight the rise of gene therapy and nanomedicine. Recent bursts (2023–2025), like “hypoxia” and “differential gene expression,” suggest a shift toward tumor microenvironment and gene regulation studies. This trend shows a progression from traditional pharmacology to gene regulation, nanomedicine, and microenvironment-focused research.

Keyword clustering grouped publications into 11 themes (**Figure 4C**). The clustering structure was evaluated using modularity Q and silhouette S metrics, yielding a Q value of 0.6676 and a weighted mean silhouette S of 0.8492, indicating a significant and highly credible clustering structure. Three core clusters dominate: #1 Drug Delivery Systems, #3 Immunotherapy, and #5 Hepatocellular Carcinoma. Peripheral clusters, such as #8 Network Pharmacology and #10 Drug Mechanism/Resistance, reflect the translation of emerging technologies and basic research to clinical applications. The field centers on HCC treatment, immunotherapy, and nanomedicine delivery, while also incorporating bioinformatics, radiotherapy, and drug resistance research.

The keyword timeline illustrates temporal evolution. Early-stage keywords (2016–2018), like “drug effects” and “chemotherapy,” emphasize pharmacological studies. Mid-stage keywords (2019–2021), such as “nanoparticle” and “immunotherapy,” indicate expansion to nanomedicine, immunotherapy, and gene regulation (**Figure 4D**). Recent keywords (2022–2025), including “Network Pharmacology,” reflect interdisciplinary approaches and novel therapies. This timeline highlights a shift from pharmacology to nanomedicine and gene therapy, and then to integrated, cross-disciplinary research, marking the transition toward precision CAR-based immunotherapy.

### 3.4 Author, country, and institution collaboration networks

**Figure 5:**
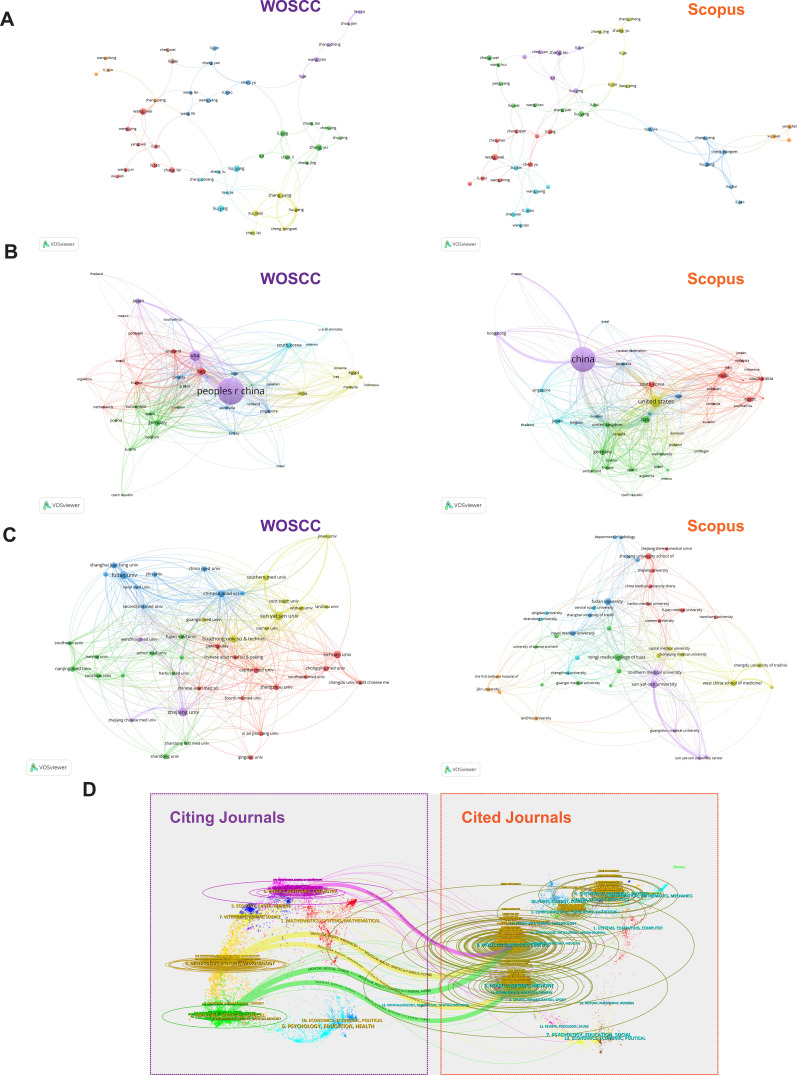
Analysis of collaborative relationships among authors, countries, and institutions: **(A)** Author collaboration network (WOSCCvsSCOPUS); **(B)** National collaboration network (WOSCCvsSCOPUS); **(C)** Institutional collaboration diagram (WOSCCvsSCOPUS); **(D)** Dual-map overlay analysis (Double Map Overlap Analysis) WOSCC.

To investigate collaboration patterns in CAR-based immunotherapy research for HCC, we analyzed the included WoSCC and Scopus publications. The author collaboration network comprised 44 high-output authors, predominantly from China (**Figure 5A**). Although WoSCC and Scopus clustered authors into 8 and 7 groups, respectively, the overall network structures were similar, indicating robust results. Despite a relatively small number of authors, the network is densely connected, suggesting the presence of a stable core group of collaborating researchers in this field.

At the country level, China dominates both research output and collaboration in WoSCC and Scopus, confirming its leading academic influence in HCC CAR research (**Figure 5B**). Other countries, including the USA, Germany, Japan, Italy, and Iran, also show substantial output and maintain strong collaborative ties.

Institutional collaboration analysis shows that active institutions are primarily Chinese universities (**Figure 5C**). Interestingly, cluster analyses in both databases reveal that institutions within the same cluster are often geographically close, indicating a preference for regional collaboration. For example, Zhejiang University clusters with Wenzhou Medical University and Zhejiang Chinese Medical University, while Shanghai Jiao Tong University, Fudan University, and the Naval Medical University form a separate cluster.

We further conducted a dual-map overlay analysis of WoSCC publications to visualize academic knowledge flows across journals and disciplines (**Figure 5D**). The left side (purple frame) represents journals and disciplines of the analyzed publications, while the right side (orange frame) represents those of cited references. The left-to-right arcs indicate knowledge flow. The analysis shows that HCC CAR research is primarily published in journals covering clinical medicine, basic biology, and engineering/materials science, while citations mainly come from clinical medicine and basic biology. Notably, materials science and computational statistics are emerging as important sources of citations, highlighting an increasing integration of medical and engineering disciplines.

### 3.5 Multiple correspondence analysis and co-citation network analysis

**Figure 6:**
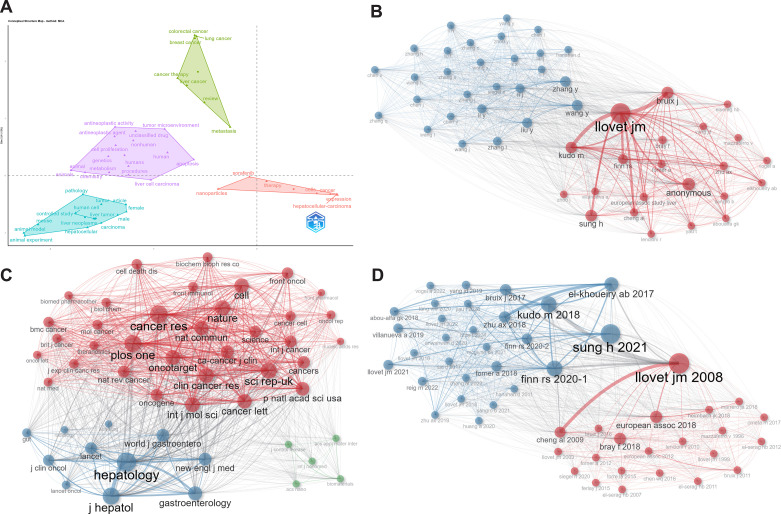
MCA, multiple correspondence analysis and co-citation network analysis: **(A)** MCA, Multiple Correspondence Analysis (WOSCC+SCOPUS); **(B)** Author Co-citation Network (WOSCC); **(C)** Journal Co-citation Network (WOSCC); **(D)** Literature Co-citation Network (WOSCC).

Multiple correspondence analysis (MCA) revealed relationships between research themes and core concepts (**Figure 6A**). Studies on “HCC” cluster in the lower-left quadrant (blue), associated with “animal experiments” and “animal models,” indicating that CAR-based immunotherapy for HCC remains largely preclinical. In contrast, the upper-right quadrant (green) covers other cancers, such as breast and colorectal, highlighting broader clinical potential. Red and purple clusters focus on “tumor microenvironment,” “metabolism,” and “chemotherapy drugs,” reflecting current basic research directions and potential combination therapies.

Author co-citation analysis identified two main clusters (**Figure 6B**). The right red cluster, centered on Llovet JM, includes international leaders such as Bruix J, Kudo M, Sung H, and Finn RS, representing global clinical and epidemiological research. The left blue cluster comprises Chinese scholars, including Zhang Y, Wang Y, Liu Y, and Chen X, focusing on CAR-T mechanisms, immune microenvironment, and molecular signaling. This dual-core structure reflects parallel international clinical leadership and domestic basic research, with growing inter-cluster citations indicating a shift toward clinical translation and global collaboration.

Journal co-citation analysis revealed three core clusters (**Figure 6C**). The red cluster (Cancer Research, *Nature*, *Science*, *Cell*) represents foundational oncology and molecular biology research. The blue cluster (Hepatology, Journal of Hepatology) reflects clinical translation in HCC immunotherapy. The green cluster (Biomaterials, ACS Nano) indicates emerging intersections of materials science and immune engineering. Dense cross-citations, especially between basic and clinical journals, highlight a multidisciplinary framework linking foundational research to clinical applications.

Finally, co-citation networks illustrate core research trajectories (**Figure 6D**). Two clusters emerge: the red cluster focuses on clinical translation, exemplified by Cheng AL (2009, *Lancet Oncol*) (25) and Llovet JM (2008, *J Natl Cancer Inst)* (26). The blue cluster emphasizes mechanistic studies and combination therapies, including Kudo M (2018, *Hepatol Res)* (27), El-Khoubeiry AB (2017, *J Clin Oncol*) (28), and Zhu AX (2018, *Lancet Oncol*) (29), providing foundational support for CAR-based immunotherapy in HCC.

### 3.6 Thematic evolution

**Figure 7:**
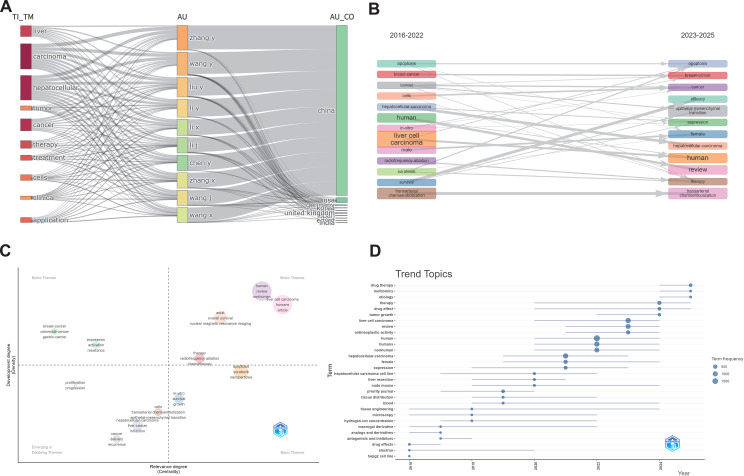
Analysis of thematic evolution trends: **(A)** Moran’s I plot (WOSCC+SCOPUS); **(B)** Thematic evolution diagram (WOSCC+SCOPUS); **(C)** Thematic centrality diagram (WOSCC+SCOPUS); **(D)** Thematic trend diagram (WOSCC+SCOPUS).

The evolution of research topics in HCC CAR-based immunotherapy can be visualized by linking major keywords, high-output authors, and their countries. The core theme centers on “HCC immunotherapy and clinical applications,” with Chinese scholars as the main contributors, consistent with previous analyses (**Figure 7A**). To track temporal trends, the study period was divided into 2016–2022 and 2023–2025 (**Figure 7B**). Keywords such as “apoptosis,” “cancer,” “HCC,” and “human” maintained high frequency across both periods, indicating a sustained focus on HCC cell death and immune mechanisms. In contrast, new keywords emerging in 2023–2025, including “epithelial-mesenchymal transition” and “efficacy,” reveal a shift toward immune microenvironment mechanisms and therapeutic evaluation. Keywords related to conventional therapies, such as “radiofrequency ablation” and “chemoradiotherapy,” have decreased, reflecting the gradual transition from traditional treatments to immunotherapy and combination strategies.

A thematic centrality map plots topics according to their network centrality (x-axis) and maturity (y-axis) (**Figure 7C**). Core and mature topics, or “Motor Themes,” such as “liver cell carcinoma,” “overall survival,” and “review/article,” indicate that CAR-related HCC immunotherapy has moved from exploratory research to clinical translation. “Basic Themes” in the lower-right quadrant, including “apoptosis,” “sorafenib,” and “nanoparticles,” represent foundational research and emerging innovations. Peripheral but mature directions, such as “breast cancer” and “colorectal cancer,” indicate the cross-cancer expansion of CAR-related research. The lower-left quadrant identifies emerging or declining topics, with “epithelial-mesenchymal transition” and “recurrence” as new directions, while traditional approaches such as “transarterial chemoembolization (TACE)” appear to be gradually phased out.

The temporal analysis of HCC CAR-based immunotherapy from 2016 to 2025 demonstrates that early studies (2016–2018) focused on HCC cell models and pharmacological mechanisms. Between 2019 and 2022, research emphasis shifted to clinical applications and patient population analyses. From 2023 onwards, emerging keywords such as “multiomics,” “drug therapy,” and “tumor growth” indicate the adoption of systems biology approaches and precision medicine strategies (**Figure 7D**). Overall, research has progressed from single-mechanism studies toward multidimensional clinical translation, forming an emerging framework focused on multi-omics integration, combination therapies, and immune microenvironment modulation.

### 3.7 Citation analysis

**Figure 8:**
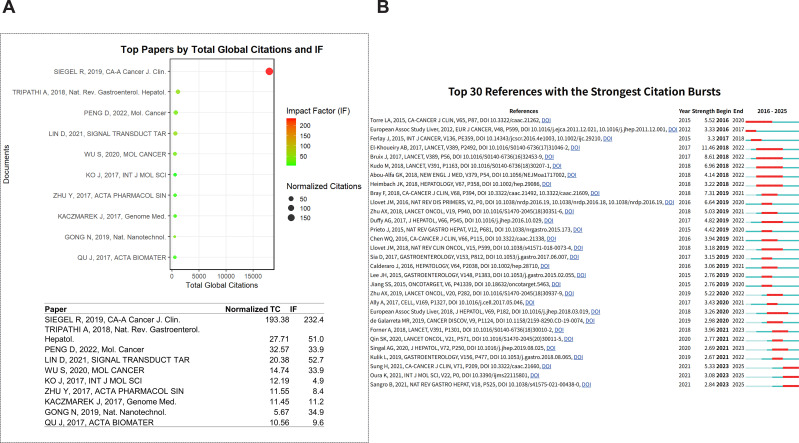
Citation analysis: **(A)** Top 10 Globally Cited Publications: Citation Counts and IF Bubble Chart (WOSCC+SCOPUS); **(B)** Top 30 Publications with Citation Bursts in WOSCC.

The most globally cited article in the field is Siegel R et al. (2019, *CA-A Cancer J Clin*) (30), with a normalized citation of 193.58 and an impact factor of 232.4, demonstrating its central role in HCC and cancer epidemiology (**Figure 8A**, **Table 3**). Other highly cited publications include Tripathi A (31) and Peng D (32), indicating their significance in HCC immunotherapy research.

**Table 3:** Global top 20 cited publications ranking and detailed information (WOSCC+SCOPUS).

Paper	DOI	Total citations	TC per Year	Normalized TC
SIEGEL R, 2019, CA-A Cancer J. Clin.	10.3322/caac.21551	18019	2,574.14	193.38
TRIPATHI A, 2018, Nat. Rev. Gastroenterol. Hepatol.	10.1038/s41575-018-0011-z	1133	141.63	27.71
PENG D, 2022, Mol. Cancer	10.1186/s12943-022-01569-x	736	184.00	32.57
LIN D, 2021, SIGNAL TRANSDUCT TAR	10.1038/s41392-021-00817-8	629	125.80	20.38
WU S, 2020, MOL CANCER	10.1186/s12943-020-01238-x	589	98.17	14.74
KO J, 2017, INT J MOL SCI	10.3390/ijms18122589	589	65.44	12.19
ZHU Y, 2017, ACTA PHARMACOL SIN	10.1038/aps.2017.5	558	62.00	11.55
KACZMAREK J, 2017, Genome Med.	10.1186/s13073-017-0450-0	553	61.44	11.45
GONG N, 2019, Nat. Nanotechnol.	10.1038/s41565-019-0373-6	528	75.43	5.67
QU J, 2017, ACTA BIOMATER	10.1016/j.actbio.2017.06.001	510	56.67	10.56
MA S, 2019, J HEMATOL ONCOL	10.1186/s13045-019-0805-7	485	69.29	5.20
LUO H, 2019, Chin. Med.	10.1186/s13020-019-0270-9	461	65.86	4.95
JIANG Y, 2019, HUM VACC IMMUNOTHER	10.1080/21645515.2019.1571892	447	63.86	4.80
CHAN A, 2018, J HEPATOL	10.1016/j.jhep.2018.08.027	416	52.00	10.17
LIU Y, 2023, J. Hepatol.	10.1016/j.jhep.2023.01.011	400	133.33	24.63
REÍTER R, 2017, Int. J. Mol. Sci.	10.3390/ijms18040843	384	42.67	7.95
LI W, 2017, Mol. Cancer	10.1186/s12943-017-0706-8	378	42.00	7.82
LOCKE W, 2019, Front. Genet.	10.3389/fgene.2019.01150	362	51.71	3.88
NIEBERLER M, 2017, CANCERS	10.3390/cancers9090116	361	40.11	7.47
ZUO X, 2020, J. Hematol. Oncol.	10.1186/s13045-019-0839-x	353	58.83	8.84

Analysis of citation bursts highlights shifts in research focus over time (**Figure 8B**). In the early stage (2016–2017), bursts primarily concentrated on HCC epidemiology and clinical guidelines, such as the 2012 EASL-EORTC HCC guideline (33) and global cancer statistics (34), reflecting the nascent stage of the field. During the mid-stage (2018–2019), bursts shifted to immunotherapy, including a 2015 review by Prieto et al. (35) and clinical trials by Bruix (36), Kudo (37), and Ghassan K (38). Notably, El-Khoueiry AB’s nivolumab trial in advanced HCC exhibited the most prominent burst (39). This period also saw rising interest in molecular targeted therapy, exemplified by Llovet JM’s review on HCC molecular therapy and precision medicine (40).

After 2020, citations increasingly focused on molecular mechanisms of drug resistance and HCC genomics, such as the 2017 *Cell* study on HCC genomic features (41). Sorting the top 30 burst-citation publications by year demonstrates a clear shift in research focus, with CAR-based HCC immunotherapy gradually moving from clinical trials toward mechanistic and translational studies.

## 4 Discussion

This study presents the first systematic and comprehensive bibliometric analysis of CAR-based immunotherapy applications in HCC from 2016 to 2025, elucidating the field’s developmental trajectory, research hotspots, global collaboration patterns, and emerging trends. By analyzing 5,210 publications from the WoSCC and Scopus databases, we provide a macro-level overview of knowledge evolution in this frontier domain.

### 4.1 Insights from bibliometric analysis

Our bibliometric analysis shows that research on CAR-based immunotherapy for HCC has expanded rapidly in recent years and is now moving into a more mature stage. The field has shifted from early exploratory work toward a broader and more integrated research landscape that includes gene engineering, tumor microenvironment regulation, biomarker discovery, and combination strategies. This pattern suggests that CAR-based immunotherapy for HCC is no longer being studied as an isolated approach, but increasingly as part of a wider effort to overcome the biological and translational barriers of solid tumors.

Another clear finding is that the current knowledge structure is still shaped mainly by preclinical studies, while clinical translation remains limited (17). The weak overlap between mechanistic research and clinically oriented work suggests that the gap between laboratory innovation and real-world application has not yet been fully bridged. At the same time, the growing role of engineering, materials science, and computational methods indicates that future progress will likely depend on multidisciplinary integration rather than on advances in immunotherapy alone.

A further notable feature of this field is the dominant role of China in publication output, together with a relatively low level of international collaboration. This pattern likely reflects a combination of epidemiological pressure, domestic capacity building, and structural barriers to cross-border cooperation. China carries a heavy burden of liver cancer, and this long-standing disease burden has created strong clinical demand for HCC-focused basic and translational research (42). In parallel, continuous investment from national funding systems has supported the rapid development of liver cancer research across basic biology, translational platforms, and therapeutic innovation. The expansion of China’s cell therapy pipeline in recent years may also have reinforced this trend, especially in the context of increasing interest in locally developed and technically self-sufficient therapeutic platforms (43).

At the same time, high publication output has not translated into equally strong multinational collaboration. One likely reason is that HCC research in China is closely linked to local patient resources, hospital networks, and disease patterns, particularly in the context of hepatitis B-related liver cancer, which remains more prominent in the Asia-Pacific region than Differences in epidemiological background may therefore shape research priorities, target populations, and clinical development strategies across regions (44). In addition, international collaboration in cell therapy still faces practical barriers, including differences in regulatory pathways, manufacturing standards, trial design, and endpoint selection. These issues are especially important in a field that depends heavily on standardized production, quality control, and translational coordination.

Data governance may also contribute to this pattern. In recent years, the regulation of human genetic resources and cross-border data transfer in China has become more structured and more closely supervised. Although these changes may strengthen governance and protect national interests, they may also increase the administrative burden of multinational research (45). Language barriers, uneven data-sharing mechanisms, and growing international competition in cell and gene therapy may further encourage domestically centered research networks. Taken together, these factors may help explain why China has become the leading contributor to this field while the level of international collaboration remains relatively limited.

### 4.2 Recent advances in CAR-based immunotherapy for hepatocellular carcinoma

Recent studies suggest that CAR-based immunotherapy for HCC is entering a more refined stage of development. While early research was largely centered on target identification and proof-of-concept validation, recent work has increasingly focused on how to improve the performance of CAR-based therapies under the biological constraints of solid tumors. In HCC, these constraints mainly include antigen heterogeneity, limited tumor infiltration, functional exhaustion in an immunosuppressive microenvironment, and the risk of on-target/off-tumor toxicity. Together, these features indicate that progress in this field is increasingly driven by engineering optimization rather than by target discovery alone (46–48).

One notable trend is the growing emphasis on engineered CAR platforms with enhanced resistance to suppressive tumor conditions. Recent reviews have highlighted that, in solid tumors, durable efficacy depends not only on antigen recognition but also on sustained persistence, signaling fitness, and resistance to inhibitory pathways within the tumor microenvironment (46, 48). In this context, the microenvironment is no longer viewed merely as an external barrier, but as a central determinant of therapeutic design. This shift is particularly relevant in HCC, where the local immune milieu remains one of the main obstacles to effective cellular therapy.

A second emerging direction is the move from single-antigen targeting toward multi-target and context-dependent recognition strategies. Because HCC often shows marked intratumoral heterogeneity, recent studies have increasingly explored dual-target or logic-guided designs to reduce antigen escape and improve tumor selectivity (46, 49). This conceptual shift reflects a broader change in the field, from static target matching toward more adaptive recognition systems that are better suited to the complexity of solid tumors (50).

Metabolic optimization has also become an important theme in recent HCC-oriented CAR research. A representative 2024 study showed that GPC3-targeted CAR-T cells engineered to express GLUT1 or AGK exhibited improved persistence, reduced apoptosis, and enhanced antitumor activity in preclinical HCC models (51). These findings support the view that metabolic fitness is an important determinant of CAR-cell function in nutrient-restricted tumor environments and further indicate that improving intracellular adaptability may be as important as improving antigen recognition itself.

Another development is the gradual expansion of the field beyond conventional CAR-T cells. Recent studies have suggested that CAR-NK-based approaches may offer additional flexibility in HCC, particularly in the context of safety optimization and locoregional or biomaterial-assisted delivery (52, 53). Although these strategies remain at an early stage, they illustrate that current advances in HCC are increasingly taking place within a broader CAR-based therapeutic framework rather than within CAR-T therapy alone.

Taken together, recent advances in HCC do not point to a single dominant breakthrough, but rather to a change in research logic. The field is moving from early target-centered exploration toward integrated optimization strategies involving enhanced persistence, adaptive recognition, metabolic support, and platform diversification. This trend is consistent with the broader evolution of CAR-Based Immunotherap in solid tumors and may define the next stage of translational development in HCC.

### 4.3 Mechanisms and adverse effects of CAR therapies

The CAR concept was first proposed in 1993 by Eshhar et al., who fused an antibody single-chain variable fragment (scFv) with the T-cell receptor signaling chain, enabling T cells to specifically recognize surface antigens (54). Subsequent optimizations have established a mature CAR structure composed of four functional modules: extracellular antigen-recognition domain, hinge/spacer, transmembrane domain, and intracellular signaling domain (55, 56).

Based on the immune cell type used, CAR-based strategies now include CAR-T, CAR-NK, and CAR-M, among which CAR-T cells have been the most extensively studied and clinically applied. Standard CAR-T production involves isolating leukocytes from patient peripheral blood, purifying T cells, ex vivo activation, CAR gene transduction via viral vectors, expansion, quality control, and reinfusion. Upon reinfusion, CAR-T cells recognize tumor antigens (e.g., CD19) through their extracellular domains, activate intracellular signaling, proliferate, and exert cytotoxic effects (57). Activated CAR-T cells release perforin, granzyme, and inflammatory cytokines, recruiting additional immune cells to mediate tumor cell killing.

Despite rapid progress, CAR therapies in HCC face notable safety challenges. HCC-specific targets such as GPC3 exhibit low-level expression in normal tissues, and GPC3-directed CAR-T therapies have been associated with liver toxicity in advanced HCC patients (58). This “on-target, off-tumor” toxicity remains a primary limitation. Next-generation designs, including logic-gated and switchable CARs, aim to preserve antitumor activity while reducing systemic toxicity, offering potential safety improvements (59).

Cytokine release syndrome (CRS) is the most common adverse event in CAR-based immunotherapy, arising from excessive T-cell activation upon antigen recognition. Activated T cells release IL-2, IFN-γ, IL-6, GM-CSF, while bystander immune cells amplify the inflammatory response. Clinically, CRS manifests as fever, fatigue, myalgia, and anorexia, and severe cases may involve multiple organ systems (60, 61). Close monitoring and interventions, such as anti-IL-6 therapy or corticosteroids, are necessary (62). Recent studies suggest *in situ* PEGylation of CAR-T cells can mitigate CRS by suppressing monocyte overactivation (63).

Neurotoxicity, termed CAR-T-related encephalopathy syndrome (CRES), often co-occurs with or follows CRS. Mechanisms are not fully understood but may involve cytokine infiltration into the CNS or direct T-cell migration (64, 65). Non-convulsive status epilepticus (NCSE) occurs in ~10% of CAR-T recipients (62), warranting prophylactic levetiracetam in high-risk patients. Multicenter trials of CD19 CAR-T (JCAR015) have reported cerebral edema–related fatalities, highlighting the need for mechanistic clarification (66).

### 4.4 Clinical trials of CAR or combination therapies

Numerous clinical trials have investigated CAR-based immunotherapy in HCC, with GPC3 being the predominant target (**Table 4**). GPC3 is overexpressed in ~70–80% of HCC cases but minimally expressed in normal tissues (67). In a phase I trial (NCT04756648), among seven evaluable patients, two achieved partial response (PR), three maintained stable disease (SD), and median overall survival (mOS) was 11.6 months (68). The TAK-102 CAR-T construct, incorporating IL-7 and CCL19, enhanced expansion and persistence of memory T-cell subsets. In another phase I trial (NCT04405778), four of eight patients achieved SD, and three showed stable or decreased alpha-fetoprotein (AFP) levels, consistent with clinical outcomes (69).

**Table 4:** Clinical trials of CAR therapy in HCC.

Targets	Indications	Regimen	Outcomes	National clinical trials identifier	Total
GPC3	HCC	CT0180 monotherapy	2/7 PR, 3/7 SD;median survival,11.6 months;	NCT04756648	7
GPC3	HCC	TAK-102 monotherapy	4/8 SD, 3/8 AFP decrease or stabilization	NCT04405778	8
GPC3	HCC	GPC3 CAR-T monotherapy	1/6 PR,3/6 SD, 2/6 PD; OS rates at 3 years, 1 year, and 6 months were 10.5%, 42.0%, and 50.3%	NCT02395250	13
GPC3	HCC	C-CAR031 monotherapy	ORR 50.0% In all DLs; ORR 57.1% In DL4; 91.7% CRS( 1 grade 3 CRS)	NCT05155189	24
CD133	14 HCC, 7 PC, 2 CRC	CART-133 monotherapy	3/23 PR, 14/23 SD. The 3-month DCR 65.2%, mPFS 5 months.	NCT02541370	23
GPC3	HCC	G3-CAR-ori2 monotherapy	3/7 PR、2/7SD、2/7PD	ChiCTR1900028121	7
GPC3	HCC	CAR-GPC3 T-cell therapy	1 PS; 2 SD;ORR 16.7%; DCR 50%; mPFS 4,2 months;mDCR 3.2 months; mOS 7.9 months	NCT03980288.	6

Other targets have also been explored. In 2018, a phase I trial investigated autologous CAR-T targeting CD133 (CART-133) in 23 patients (14 HCC, 7 pancreatic cancer, 2 colorectal cancer), with three PRs, 14 SDs, a 65.2% three-month disease control rate, and median progression-free survival (PFS) of 5 months (NCT02541370). Trials targeting AFP are ongoing (NCT03132792), with anecdotal reports indicating potential synergy with targeted therapies (70).

Overall, early-phase trials demonstrate promising efficacy for GPC3-targeted CAR-based immunotherapy in HCC. However, trials targeting other antigens or CAR modalities have yet to show substantial clinical benefits, and studies combining CAR-based immunotherapy with radiotherapy, targeted therapy, or other immunotherapies remain preliminary.

### 4.5 Future research directions and technological potential

Although CAR-T immunotherapy has demonstrated promising efficacy in HCC, significant technical and clinical challenges remain (53, 71). Future investigations are expected to advance along several complementary avenues. Multi-target and logic-gated CAR designs may be particularly important because they could better address tumor heterogeneity and immune evasion while reducing “on-target, off-tumor” toxicity. In addition to this general direction, future studies may benefit from prioritizing more specific GPC3-centered dual-target strategies. Among the combinations that appear most worthy of further evaluation, GPC3 + EpCAM may improve coverage of stem-like or progenitor-like tumor cell subsets, which are closely linked to recurrence, resistance, and intratumoral heterogeneity in HCC. Recent work also supports the broader value of dual-target designs such as FAP/GPC3, which may help limit antigen escape and better address stromal and tumor-cell heterogeneity. By comparison, GPC3 + ASGR1 may be better viewed as a more exploratory safety-oriented direction. Because ASGR1 is strongly associated with liver-specific biology, this combination may be useful in AND-gate CAR systems as a contextual restriction strategy to improve spatial selectivity and reduce unintended activation in non-tumor tissues. At present, this approach should be regarded as a priority for future validation rather than an established clinical route (50, 72–74). Second, emerging cellular platforms such as CAR-NK and CAR-M offer enhanced safety profiles and immediate effector functions, serving either as adjuncts to or in combination with conventional CAR-T therapies (53). Moreover, modulation of the tumor microenvironment and combination treatment strategies are likely to become central, with interventions targeting immunosuppressive cell populations (e.g., MDSCs, TAMs) and metabolic reprogramming pathways potentially enhancing CAR-based efficacy in solid tumors.

From a technological perspective, integrative multi-omics approaches coupled with artificial intelligence-driven target discovery are poised to accelerate the development of precision CAR-T therapies, enabling individualized treatment designs. Recent advances in single-cell and spatial transcriptomic profiling have made it possible to map antigen expression at much higher resolution across tumor cells, stromal compartments, and local immune niches in HCC. On this basis, future studies may use deep learning models, including Transformer-based frameworks, to identify novel tumor-specific antigens or prioritize antigen combinations that are more stable and selective in HCC. This may be particularly valuable because currently studied targets such as GPC3 and AFP often show heterogeneous expression across patients and tumor subpopulations. In practical terms, AI in this setting should not be limited to general prediction. A more useful next step would be to combine HCC-specific multi-omics data with spatial localization and immunogenicity-related features to rank candidate target pairs before experimental testing (75, 76). AI may also contribute at the level of safety assessment. One important future application is the development of off-target toxicity prediction models that integrate normal tissue expression atlases, protein sequence or structure similarity, and antigen-binding features before final CAR construction. Such models may help identify high-risk binders earlier in the design process and allow unsafe constructs to be removed before preclinical escalation. This direction is also consistent with recent efforts to improve CAR safety assessment through more systematic on-target and off-target evaluation workflows (77). Gene-editing techniques and controllable switchable CAR systems may further improve both safety and therapeutic stability (78–80). Facilitating multinational, multicenter clinical trials alongside the integration of real-world evidence will be critical for rapid validation and clinical translation of these advanced strategies. Collectively, future research in HCC CAR-based immunotherapy is likely to evolve from single-mechanism studies toward multidimensional, interdisciplinary, and precision-guided clinical applications, ultimately providing patients with safer and more effective personalized therapeutic options.

### 4.6 Limitations

This study has several limitations. First, inclusion criteria differ between WoSCC and Scopus; WoSCC applies stricter screening, favoring early literature and robust citation network analysis, while Scopus covers more document types, including preprints, but is less compatible with citation software. Second, data retrieval was current through September 2025, and full-year data were unavailable. Third, non-English publications were excluded, although this proportion was small. These factors do not materially affect the conclusions. Notably, bibliometric analyses capture macro-level trends and patterns but cannot replace expert evaluation of specific technical content or clinical evidence.

## 5 Conclusion

CAR-based immunotherapy has established a highly active and rapidly evolving research paradigm in HCC, with China as the central driver of global research output. Research focus has gradually shifted from early evaluation of conventional drug efficacy to frontier investigations characterized by interdisciplinary integration, encompassing immune modulation, nanodelivery, genetic engineering, and bioinformatics. Despite substantial progress in basic research, efficient translation into clinical practice remains a key bottleneck and will continue to shape future research agendas. Strengthening multicenter, international collaborations, systematically addressing safety and efficacy challenges in solid tumor immunotherapy, and accelerating engineering-driven next-generation CAR clinical validation may enable the field to transition from mechanistic innovation to clinical paradigm transformation, offering HCC patients transformative therapeutic options.

## Data Availability

The raw data supporting the conclusions of this article will be made available by the authors, without undue reservation.
