# Supplementary material for: Current progress and emerging trends of car-based immunotherapy for hepatocellular carcinoma: a data-driven atlas from multidatabase integration
Source: Front Immunol. 2026 Apr 22;17:1778197. doi: 10.3389/fimmu.2026.1778197 (PMC13143984; doi:10.3389/fimmu.2026.1778197)
Supplement: Supplementary file 1 [file SupplementaryFile1.docx]

| **Database** | **Search Strategy** | **Search Date** | **Filtering Conditions** |
| --- | --- | --- | --- |
| **WOSCC** | TS1=("CAR*" OR "Chimeric Antigen Receptor");  TS2=("Hepatocellular Carcinoma" OR "HCC" OR "Liver Cancer");  TS3=("Immunotherapy" OR "CAR-T therapy" OR "Therapy" OR "Treatment");  TS4=("Application" OR "Clinical application");  TS=TS1 AND TS2 AND TS3 AND TS4 | The literature search was conducted on October 1, 2025. | English-language publications from January 1, 2016, to September 30, 2025, including Articles and Review Articles. |
| **Scopus** | (TITLE-ABS-KEY(("CAR*" OR "Chimeric Antigen Receptor")) AND TITLE-ABS-KEY(("Hepatocellular Carcinoma" OR "HCC" OR "Liver Cancer")) AND TITLE-ABS-KEY(("Immunotherapy" OR "CAR-T therapy" OR "Therapy" OR "Treatment")) AND TITLE-ABS-KEY(("Application" OR "Clinical application"))) | The literature search was conducted on October 1, 2025. | English-language publications from January 1, 2016, to September 30, 2025, including Articles and Review Articles. |

**Supplementary Table 1. Detailed search strategies used for bibliometric analysis in WOSCC and Scopus.**

**Supplementary Table 2. Detailed search strategies for identifying clinical trials targeting GPC3 and CD133 in hepatocellular carcinoma (WOSCC and PubMed).**

| **Database** | **Search Strategy** | **Search Date** | **Filtering Conditions** |
| --- | --- | --- | --- |
| **WOSCC** | TS1 = ( "chimeric antigen receptor*" OR "CAR-T" OR "CAR T" OR "engineered T cell*" OR "modified T cell*" )；  TS2 = ( GPC3 OR "glypican-3" OR CD133 OR PROM1 )；  TS3 = ( "hepatocellular carcinoma" OR HCC OR "liver cancer" OR malignan* OR "solid tumor*" )；  TS = TS1 AND TS2 AND TS3 | The literature search was conducted on October 1, 2025. | Publications from January 1, 2016, to September 30, 2025.No language restrictions.No limitations on document type (e.g., articles, reviews, conference proceedings, letters, etc.). |
| **PubMed** | ( "chimeric antigen receptor"[Title/Abstract] OR CAR[Title/Abstract] ) AND ( T cell*[Title/Abstract] OR NK cell*[Title/Abstract] OR macrophage*[Title/Abstract] ) AND ( "Carcinoma, Hepatocellular"[Mesh] OR "hepatocellular carcinoma"[Title/Abstract] OR HCC[Title/Abstract] OR "liver cancer"[Title/Abstract] ) AND ( GPC3[Title/Abstract] OR "glypican-3"[Title/Abstract] OR CD133[Title/Abstract] OR PROM1[Title/Abstract] ) | The literature search was conducted on October 1, 2025. | Publications from January 1, 2016, to September 30, 2025.No language restrictions.No limitations on document type (e.g., articles, reviews, conference proceedings, letters, etc.). |

**Supplementary Table 3. Inclusion and Exclusion Criteria for Clinical Trials of GPC3- or CD133-Targeted CAR-T Therapy in Hepatocellular Carcinoma.**

| **Category** | **Criteria** |
| --- | --- |
| **Inclusion Criteria** | 1. Study type: Only registered interventional clinical trials were included, specifically Phase I or Phase II studies with a valid NCT or ChiCTR registration number.2. Study population: Patients with primary hepatocellular carcinoma (HCC), or patients with advanced solid tumors that included an HCC subgroup.3. Intervention: CAR-T cell therapy targeting GPC3 or CD133, administered as monotherapy.4. Outcome measures: Studies reporting concrete efficacy data (e.g., objective response rate, disease control rate, progression-free survival, or overall survival) and safety data (e.g., cytokine release syndrome or other adverse events). |
| **Exclusion Criteria** | 1. Intervention: Studies involving non-CAR-T therapies, CAR-T therapies targeting antigens other than GPC3 or CD133, combination therapies, or non-monotherapy regimens.2. Study type: Retrospective studies, animal or in vitro experiments, reviews, meta-analyses, case reports, or studies lacking original quantitative data.3. Data completeness: Studies without a clinical trial registration number, or with missing or non-extractable key data, were excluded. |

**Supplementary Table 4.** **Classification of Randomly Sampled Studies Based on Methodological Rigor and Evidence Strength.**

| **Level** | **Definition** | **Assessment Criteria** |
| --- | --- | --- |
| **A (High Quality)** | Methodologically rigorous with strong evidence | 1. Clearly stated study design and methodology; 2. Transparent data sources and appropriate statistical methods; 3. Conclusions fully supported by data, with no major bias. |
| **B (Moderate Quality)** | Generally complete information with minor limitations | 1. Study methods incompletely described or small sample size; 2. Minor reporting deficiencies that do not affect core conclusions; 3. Limitations not fully discussed. |
| **C (Low Quality)** | Methodological flaws and limited evidence | 1. Lack of clear study design or supporting data; 2. Conclusions overly speculative; 3. Or clearly outdated review/opinion articles. |

**Supplementary Table 5.** **Quality Assessment of Randomly Sampled Included Studies.**

| **Quality Level** | **Brief Assessment Criteria** | **Sample Size (n=1042)** | **Proportion** |
| --- | --- | --- | --- |
| **A (High Quality)** | Methodologically rigorous with strong evidence | 812 | 77.90% |
| **B (Moderate Quality)** | Generally complete information with minor limitations | 185 | 17.80% |
| **C (Low Quality)** | Methodological flaws and limited evidence | 45 | 4.30% |
| **Total** | — | 1,042 | 100% |
